# Pediatric cancer registries in Latin America: the case of Argentina’s pediatric cancer registry

**DOI:** 10.26633/RPSP.2017.152

**Published:** 2017-12-05

**Authors:** Johanna Michelle Fedorovsky, Luis Gabriel Cuervo, Silvana Luciani

**Affiliations:** 1 American University American University Washington, D.C. United States of America American University, Washington, D.C., United States of America.; 2 Pan American Health Organization Pan American Health Organization Washington, D.C United States of America Pan American Health Organization, Washington, D.C., United States of America.

**Keywords:** Child health services, neoplasms, surveillance, Latin America, Argentina, neoplasias, vigilancia, América Latina, Argentina, de da criança, neoplasias, vigilância, América Latina, Argentina

## Abstract

*Despite its low incidence, pediatric cancer makes up a significant portion of childhood illnesses. Yet information on pediatric cancer in Latin America is scarce. Since the early 2000s the World Health Organization (WHO) has been highlighting the role of cancer registries in cancer surveillance and control. This article describes the main aspects of pediatric cancer registration in Latin America, highlighting the successes of Argentina’s national pediatric cancer registry, Registro Oncopediátrico Hospitalario Argentino (ROHA), which allows for better health care and contributes to improved outcomes for children with cancer, to provide a rationale for the expansion and enhancement of pediatric cancer registration in other Latin American countries*.

## RESUMEN

A pesar de tener una incidencia baja, el cáncer pediátrico representa una proporción significativa de las enfermedades de la niñez. Sin embargo, en América Latina hay poca información al respecto. Desde comienzos del siglo XXI, la Organización Mundial de la Salud (OMS) ha puesto de relieve la función que tienen los registros oncológicos en la vigilancia y el control del cáncer. En este artículo se describen los principales aspectos de los registros de cáncer pediátrico en América Latina y se pone el acento en los éxitos del Registro Oncopediátrico Hospitalario Argentino (ROHA), que permite mejorar la atención de salud y ayuda a obtener mejores resultados en los niños con cáncer, lo cual puede servir de fundamento para la ampliación y el perfeccionamiento de los registros de cáncer pediátrico en otros países latinoamericanos.

## RESUMO

Apesar da incidência baixa, o câncer em crianças representa uma parcela considerável das afecções infantis. Porém, existem poucos dados sobre o câncer em crianças na América Latina. Desde o princípio dos anos 2000, a Organização Mundial da Saúde (OMS) trabalha para reforçar o papel dos registros de câncer no controle e vigilância da doença. Este artigo examina as principais características dos registros do câncer em crianças na América Latina e põe em relevo os bons resultados obtidos com o registro nacional da Argentina (Registro Oncopediátrico Hospitalario Argentino, ROHA), como melhoria da assistência de saúde e dos desfechos aos pacientes pediátricos, servindo de base para a ampliação e o aperfeiçoamento do registro de câncer em crianças em outros países latino-americanos.

Pediatric cancer refers to a group of cancers affecting children between 0–14 years of age (or, when adolescents are included in this classification, between 0–19 years old). Current incidence rates are low, ranging from 50 to 200 cases per million per year, depending on the country (1). However, with the advances in the control of communicable diseases, pediatric cancer is gaining importance as a childhood illness. The International Classification of Childhood Cancer (ICCC) (2) lists 12 main categories of cancers. Leukemia and lymphoma are the most common types in the world, followed by brain and other central nervous system tumors (1). In high-income countries (HICs), overall pediatric cancer five-year survival rates are 80% or higher (1). For lymphoblastic leukemia, five-year survival exceeds 90% in countries such as the United States and Germany (3, 4). These outcomes are the reason why pediatric cancer control in these countries can be considered a “success story” (1) and a marker of their high-quality health services. Survival data for low- and middle-income countries (LMICs) are not widely available, but the estimates suggest more modest results compared to HICs (1, 5). The results are highly heterogeneous among LMICs, including five-year survival rates that range from 5% to 60% (4).

The gap in survival between HICs and LMICs emerges from differences in available treatments; health resources; infrastructure; early detection; clinical support, and follow-up; and coexisting malnutrition and communicable diseases (5, 6). One strategy that LMICs could adopt to deal with some of these issues is to strengthen their pediatric cancer registration mechanisms to cover the totality (or at least a significant percentage) of pediatric cancer cases within their borders. Reports published by the World Health Organization (WHO) in 2002 and 2011 stress that population-based cancer registries are a key feature of national cancer programs (7, 8). In line with that, the Pan American Health Organization (PAHO) Policy on Research for Health (2009) underscores the need to develop registries in Latin America (9). Expanding pediatric cancer registration in Latin America could serve to better inform national policies. It would also allow for more thorough regional and international comparisons, helping to identify unjustified variations in treatment response and survival.

Latin American countries need to strengthen their cancer registration mechanisms to meet good-quality criteria for completeness, coverage, and timeliness of data (10–13). Cancer registration—for both adult and pediatric cases—does not have a long history in the region. The first good-quality population-based cancer registry in Latin America was created in 1962 in Cali, Colombia (14, 15). Many of the cancer registries created since then have an institutional or local scope rather than national coverage, limiting their potential to inform health public policies. That is the case of local registries in Argentina, Brazil, Chile, Colombia, Ecuador, Honduras, and Peru. Only Argentina and Chile have national pediatric cancer-specific registries (16). Argentina’s national pediatric cancer registry, the Registro Oncopediátrico Hospitalario Argentino (ROHA) (17), was created in 2000. It was the first Latin American pediatric cancer registry with a national scope. Nowadays, ROHA is expanding its coverage and developing new features, such as real-time online patient registration. In addition, ROHA’s staff provides technical cooperation to other countries in the region for the creation or improvement of pediatric cancer registries.^a^

This article describes the main aspects of pediatric cancer in Latin America, highlighting the successes of ROHA, which allows for better health care and contributes to improved outcomes for children with cancer, to provide a rationale for the expansion of pediatric cancer registration in the region.

## GENERAL CONSIDERATIONS ABOUT CANCER REGISTRIES

Registries enable the surveillance of cancer through the ongoing, timely, and systematic collection of information on new cancer cases, treatments, and survival and deaths rates (18–20). The registries allow for better planning of cancer policies and are valuable tools for the monitoring and evaluation of the effectiveness and efficiency of the health system. Cancer registries are usually categorized in two main groups: hospital-based (HB) and population-based (PB). HB registries focus on the patients of a single institution and can provide detailed data to inform administrative decisions as well as clinical epidemiology. PB registries are the gold standard for cancer registration as they cover a well-defined population in a geographic area instead of focusing on one institution. Some of the essential variables of PB registries are sex, date of birth, incidence date, location of the tumor, and tumor morphology. PB registries gather data from multiple sources, including pathology laboratories, death certificates, and autopsy reports (19).

Building and running a pediatric cancer registry with a population scope is challenging, especially in limited-resource settings (19). Nevertheless, the low volume of pediatric cancer cases makes it possible to create a high-quality national registry, especially in countries where treatment of pediatric cancer is concentrated in specific hospitals. The case of ROHA is a reflection of that.

## PEDIATRIC CANCER REGISTRATION IN ARGENTINA: THE CASE OF ROHA

### Main features

ROHA was the first nationwide pediatric cancer registry in Latin America. It was created within the framework of the Kaleidos Foundation (21), an Argentine nongovernmental organization, following international standards (22) and the example of well-established pediatric cancer registries such as the German Childhood Cancer Registry (GCCR) (23). In 2010, the National Cancer Institute of Argentina (Instituto Nacional del Cáncer, INC) was created, and ROHA began operating as part of the institute. The Argentine government now provides funding and an institutional framework to ROHA. Since its inception, ROHA has been gathering information from all institutions where children with cancer are cared for, thus offering national coverage of pediatric cancer cases (23 provinces and the capital district). The estimated coverage of ROHA is 90% (with the remaining 10% corresponding to data losses) (24).

One salient feature of ROHA is that it combines the characteristics of PB and HB registries in order to ensure national coverage. As the treatment of pediatric cancer is concentrated in highly specialized facilities in Argentina, it is possible to use hospitals as the basic unit of the registry. ROHA complements the information that hospitals provide with other relevant sources, such as private clinics, local population registries, death certificates, pathology laboratories, and radiotherapy centers. The doctors treating pediatric cancer patients are usually in charge of the registration, but specially trained administrative personnel can also fulfill this task. ROHA coordinates the network, provides support, and compiles the data. Its permanent staff includes a director, an epidemiologist, a systems analyst, and two coders trained to codify cases according to the ICCC. As of 2015, the network included 88 institutions (24).

ROHA’s database contains information about type of tumor, incidence, place/s of treatment, survival, and mortality (24). The National Identification Number (Documento Nacional de Identidad, DNI), a unique number assigned by the Argentine government to citizens and residents, serves to identify patients. The DNI and other personal data of the patients are treated as confidential by ROHA. Data can be aggregated at different levels: national, provincial, departmental, and hospital. ROHA reports its data on average every two years in publications that can be accessed free of charge either online or in print. Stakeholders can request data that are available but unpublished. ROHA provides more detailed pediatric cancer information than most registries in Latin America, which typically only include data about incidence, lack a national scope, and rely on projections.

### The relevance of ROHA for decision-making

ROHA provides crucial data for the identification and development of lines of action for public policies on pediatric cancer in Argentina. The availability of high-quality data is important for developing, implementing, and assessing pediatric cancer strategies. The recently published guideline “Pediatric Central Nervous System (CNS) Tumors” (25) and the promotion of early diagnosis of pediatric cancer in Argentina are two examples of public policies informed by ROHA’s data.

**Pediatric CNS tumor guidelines.** ROHA’s data on CNS tumors highlighted the need for pediatric CNS tumor guidelines by identifying unjustified variations in survival outcomes across different provinces and an average national survival rate considerably lower than those observed in other HICs (Table 1). The INC developed the guidelines (25) to standardize pediatric CNS tumor management, promote best practices, and create an environment where departures from standard treatment require a substantiated motive. Based on both evidence and expert consensus, the guidelines provide a rationale for centralizing surgical interventions in hospitals with greater levels of expertise and more skilled teams.

**Promotion of early diagnosis.** ROHA’s information illustrated that many cases of pediatric cancer were being reported at late stages. This triggered an initiative to train primary care providers in the early signs and symptoms of childhood cancer in order to reduce these delays in diagnosis. Trained staff members from the INC regularly travel to Argentine provinces to conduct early diagnosis workshops and to learn from the situation in the field (26). The staff members train medical personnel and trainers so that the workshops can be replicated throughout the provinces. In 2016, workshops were held in seven provinces in different regions of Argentina.^b^ ROHA’s data informed the selection of provinces, with priority given to those with greater pediatric cancer incidence rates and/or more advanced stages of cancer at diagnosis.

### The future of pediatric cancer registration in Argentina and across Latin America

For the past two years, ROHA has been using online registration to collect real-time information on patients. The development of the open code online registration software cost US$ 15,000.b Since its implementation, 71% of patients have been registered online. ROHA’s goal is to achieve a 100% online registration by the end of 2017. It also aims to expand the network of hospitals that provide information to the registry, thereby increasing the national population coverage beyond the current 90%.

In the Americas region, technical cooperation between countries with similar challenges and resources could be strategic in boosting good-quality cancer registration. Countries could learn and build upon existing experiences while using some of the capacities and tools already developed. The International Agency for Research on Cancer (IARC) (Lyon, France) has created a cancer registry training hub at the INC to stimulate collaboration and improve cancer registries in the region. Within this context, registries like ROHA could be developed and adapted to local conditions to expand the number of pediatric cancer registries in Latin America. Florencia Moreno, director of ROHA and member of the IARC’s “International Incidence of Childhood Cancer” editorial board (27), stated that this could be an auspicious moment for the promotion and expansion of pediatric cancer registration in Latin America.^c^ She highlighted the ongoing collaboration between ROHA and teams in Paraguay and Peru to help the two countries develop their own registries. While these and other projects continue to grow, it is equally important to continue developing the cancer registries that already exist and increase their visibility and use. For instance, ROHA could have a website offering access to its datasets so that users could conduct their own analysis of the information. It would also be useful to provide information about the registries’ cost-effectiveness; registries like ROHA could, for example, maintain records of the policies they have contributed to and report the information to the public.

**TABLE 1:** Five-year survival rates for 1) all tumors and 2) central nervous system (CNS) tumors, Argentina (2000–2009) and Germany (2005–2014)

Type of tumor	Five-year survival rate (%)	
	Argentina	Germany
All	61	85
CNS	44	77

***Source:*** Prepared by the authors with data from the Registro Oncopediátrico Hospitalario Argentino (ROHA) (24) and the German Childhood Cancer Registry(4).

## CONCLUSIONS

Pediatric cancer registries with population coverage are essential tools for monitoring and evaluating the performance of cancer treatments and health systems, tracking progress, and providing evidence for timely corrective actions. In Latin America, cancer registration in general, and pediatric cancer registration in particular, are still in the development stage and could benefit from lessons learned and benchmarks drawn from innovative registries like ROHA. The ongoing technical cooperation between Latin American countries allows for optimism regarding the future of pediatric cancer registration in the region. However, a long-term commitment from each Latin American country will be necessary for these initiatives to thrive.

### Acknowledgments

Florencia Moreno (Registro Oncopediátrico Hospitalario Argentino (ROHA)/Instituto Nacional del Cáncer (INC)) provided key inputs for this article through several interview sessions. Louisa Stüwe and Ana Cristina Castro (PAHO/WHO) and Nicole Corea (American University) reviewed the article and contributed valuable suggestions.

### Disclaimer

Authors hold sole responsibility for the views expressed in the manuscript, which may not necessarily reflect the opinion or policy of the RPSP/PAJPH, or the Pan American Health Organization (PAHO).

## Footnotes

a Personal interview with Florencia Moreno (ROHA and Programa Nacional de Cuidado Integral del Niño y Adolescente con Cáncer (PROCUINCA) / INC, Buenos Aires), July–August 2016.

b Personal interview with Florencia Moreno (ROHA and Programa Nacional de Cuidado Integral del Niño y Adolescente con Cáncer (PROCUINCA) / INC, Buenos Aires), July–August 2016.

c Personal interview, July–August 2016.
